# The Homoiopathic Doctrine

**Published:** 1832-07-01

**Authors:** 


### VIII.

### La Doctrine Homoiopathique. Par *Hahnemann.*

Readers, are you in search of truth? for if ye are, incline your ears to the oracles of wisdom in this wonderful book. Truth, M. Hahnemann assures us, is eternal as the Divinity: men may neglect it for a season, but the time inevitably arrives that it will pierce through the clouds of prejudice, and diffuse over mankind a beneficent light, which cannot be obscured. Such is the appropriate exordium to the revelation of his most magnificent discovery of homoiopathia, or the mode of curing one disease by giving another which is like to it, and which, being more powerful, is to overwhelm and extinguish the former. Strange that men, since the days of Adam, have been so hard of perception and of understanding, as not to have found out this most simple and sublime axiom of science, that the only method of annihilating any sensation, either of joy or of pain, is to drown it in one that is more intense! How is it, exclaims M. Hahnemann, that we cannot see Jupiter in the morning sky? Simply because it is hid by the superior splendour of the sun. How do we try to stifle any offensive odour? By snuffing tobacco, which has a stronger one. How shall we drown the lamentations of the miserable? but by the noise of fifes and drums. Is not the kettle-drum intended to deafen the roar of the distant cannon to the ear of the soldier? and is it not the best way of preventing the bad effects of excessive joy, to drink a dish of coffee, which, M. H. informs us, disposes the soul to most agreeable and happy impressions? But, in order to place this most important truth still more prominent, as far as regards medicine, we shall avail ourselves of a few, out of the many professional proofs adduced by our author, in confirmation of his doctrines:—Cholera morbus may be cured by giving drastic purges, as the white hellebore!—Profuse sweating is best counteracted by sudorifics!—Vertigo and nausea by narcotics such as tobacco!—Ophthalmia may be cured by acrid applications.—Dropsy by means which, according to the German physicians, will cause it in a healthy person.—Bronchitis by stimulating expectorants.—Dyspepsia, by such herbs as cause an extreme exhaustion of the vital energies, render the digestion painful, and destroy the appetite.—Inflammatory fevers by generous wines.—Drowsiness by the exhibition of opium.—Menorrhagia by the savin powder.—Strangury by cantharides.—Asphyxia by inhaling the fumes of sulphur.—Cancer by arsenic, which, M. Hahnemann assures us, often gives rise to cancer.—Inflammation of the tongue and fauces by mercury. Do we wish to cool a patient? immerse him in a warm bath. Does he labour under the effects of a coup de soleil? foment his head with hot decoctions. Has he diabetes? give him diuretics—or are his stomach and bowels inflamed? assuredly nothing is so serviceable as small doses of corrosive sublimate. We might easily enlarge our illustration; but the truth must now, we hope, be so very obvious and convincing to our readers, as to preclude the necessity.

Let us only keep in mind, therefore, that the groundwork of the theory is, that the deranged state of the organization, or of the function of a part, which is called disease, cannot be brought to a state of health, except by exciting another and a similar disease by the aid of medicines. Such is Hahnemann's homoiopathia, the only method which leads us, in a straight line, to an agreeable, certain, and lasting cure without causing any injury, or at all weakening the patient. The other two methods of treating diseases, and those which have been pursued by all, before this great German luminary appeared in the medical horizon, are designated by our author "allopathia," or "heteropathia," and "antipathia," or "enantiopathia;" under the former is classed the employment of bleeding and of blisters in inflammations; the use of purgatives and diaphoretics in fever; of diuretics in dropsy, and so forth; and, in short, any endeavour to check, control, or modify any morbid action of the sanguiferous, absorbent, and nervous systems, by exciting an action at variance with the existing malady, or by assisting Nature in any effort which she makes to relieve herself of it; whereas the latter denotes the practice of prescribing for insulated symptoms—as of ordering opium against watchfulness, laxatives against constipation, cold applications against burns, warm drinks against shivering, generous food and drink against weakness; a practice, according to the doctrines of the homoiopathic school, at once deceitful in its apparent effects and dangerous in its consequences. After these few words of prefatory explanation, we proceed rapidly to review the different sections of the work before us; to criticise it must be quite unnecessary, as there cannot be much difference of opinion on the score of its merits.

We much regret that the Doctor has not treated his readers, as he does his patients, by homoiopathic doses as minute and concentrated as possible; had he but docked one cipher from the 500 pages of this great work, his mode of treatment would have been as successful, and assuredly much more pleasant. We often have thought, while perusing the volume, of that sarcasm of D'Alembert —"an author bothers himself to amplify, what his reader equally bothers himself to abridge."

The first fifty pages are occupied with an assault and battery of all the ordinary common-sense doctrines of disease, which have prevailed in the world till Hahnemann propounded his views—views, says he, which are far elevated above that mechanical routine, of endeavouring to trace the causes and essential characters of maladies with a view to their cure. Hitherto it has been rather a boast of the enlightened members of our profession, to examine and watch carefully the operations of Nature; what means she employs, and what course she follows, in attempting to relieve herself of, or to repair, any injury done to the body; and we have been so blindly rash as to attribute much of our supposed superiority to the simpler and more rational views thence deduced. But it now appears that we have been led by an ignorant and foolish guide. Listen to the opinion of Dr. H.:—" Nature is brutish, and fails in almost all the efforts she makes: she cannot reason—she is incapable of reflection—she cannot, like a skilful surgeon, close the lips of a wound, and heal them by the first intention; she cannot cure an oblique fracture, however much callus she throws out round the broken ends of the bone; she cannot tie a bleeding artery; she cannot reduce a dislocation; and if permitted to have her own way, will even be so perverse as to make it irreducible;—so stupid is she, that in order to relieve herself from a foreign body impacted in the cornea, she will destroy the eye by suppuration; and in a strangulated hernia, she knows no other mode of overcoming the imprisonment, but by gangrene and death. In opposing any acute malady, her operations are directed by the laws of mere corporeal organization, and not by the inspirations of thought and reflection; in short, says the Doctor, they are, in such cases, a good sample of allopathia!! In a word, every thing which she does to cure the diseases of the body, must satisfy the mind of the philosopher that her methodus medendi is a tissue of suffering and pain, and that, therefore, if he wishes to practise medicine with credit and honor, he must cast away the guidance of such an ignorant and obstinate conductress; he must abandon the practice of counteracting disease, by manfully opposing its cause or symptoms, for the oracle of Germany assures him that, even though he cures it apparently, the primary affection remains untouched and unreached, and that the fire is only smothered, to break out with redoubled fury. Let us take the case of a full, plethoric, and luxuriously-living man being attacked with gout, and consider what Nature suggests, and what physicians, hitherto trusting to these suggestions, recommend. Do we not find that the patient is feverish and thirsty—he has lost his appetite, and betakes himself to simple, cooling beverages, thus indicating that the rich viands, and exciting wines have lost their relish; the medical man when summoned will probably follow a similar course; he will restrict his patient to the lightest and simplest food; he will purge him well, and lower his strength, and may even order cool applications to the torturing and heated joints; in a few days the patient is better; and in a few more he is quite well under this very simple regimen, which is in accordance with the principles of allopathia. Well the patient may be pleased, and the doctor may boast of the accuracy of his diagnosis, and of the success of his remedies; but the joy of the one and the pride of the other, says Dr. Hahnemann, will be turned into grief and disappointment:—the disease may be broken, but is not vanquished; the strength is impaired by the bleedings, and purging and low diet, and in spite of them all, the germ of the evil remains behind. As well might an army boast of a victory, when instead of attacking an enemy in front with equal weapons, and ending the dispute by death, it resorted to the ignoble stratagem of setting fire to the country in their rear, and cutting off all possible retreat; and even although an advantage is gained, the foe may be defeated, but is not annihilated; and he only requires fresh men and more arms, to burst out with more impetuosity than ever. Such is the sublime comparison and such the irresistible (at least in his own opinion) argument, by which the Doctor demolishes the doctrines of allopathia, which have taught us to employ antiphlogistic and depletory remedies in acute, and derivative and counter-irritant ones, in chronic diseases. We may remark, that in order to be able to appreciate fully the cogency of the reasoning, it is necessary to surrender all scepticism. We must implicitly receive, as gospel, whatever the Doctor is pleased to reveal, and most carefully shut the eyes, both of our bodies and of our minds, against any opposing facts. One example will be sufficient to demonstrate this. Most people have observed that chronic ailments have frequently been much relieved and even cured by spontaneous sweating, or purging; and have imitated the practice with what they deemed success; but M. Hahnemann tells him downright that this practice must invariably have a contrary effect, and encrease ten-fold the primary disease.

Readers, we caution you to beware of contradiction; if capable of an atom of such a heresy, ye are unworthy to be disciples of the great medical reformer!! But yet how can we fail to express our surprise that a single patient labouring under any disease, should ever have recovered under the murderous system of treatment which has from the earliest ages been adopted; and that the world has not by this time become altogether tenantless. Even when we have witnessed almost immediate good effects of any remedy, we now find that it has been nothing but a cheat, and a lie of our fancy. Millions of facts, exclaims the mighty German, prove what lamentable results have flowed from giving opium against diarrhœa, sleeplessness, and chronic pains; effervescing draughts against vomiting; astringents against menorrhagia; refrigerants and acids against feverish heat; and from employing ointments made with lead and zinc in cutaneous eruptions; or even from plugging the nostrils in epistaxis. Such modes of treatment are at utter variance with the principles of homoiopathia, which is truth itself.

Another great error of the vulgar school of medicine is that of employing wine, bark and other tonics, for the purpose of strengthening a patient debilitated from a chronic disease. Instead of good, they inevitably and necessarily must do mischief!! Cures indeed have been reported, but to use the sarcastic sneer of our author, they were cures, which were of no benefit to the patient.

After this it is to be hoped that we never hear more of allopathic treatment, or as it is expressed in Latin, of trying to cure "contraria contrariis."

Sometimes indeed mere chance has directed medical men to a speedy and durable cure of some diseases; but little did they know all the time that they were employing homoiopathic remedies. What are generally allowed to be specifics, as mercury in syphilis, bark in ague, arnica in the effects of contusion, and sulphur in scabies, Dr. Hahnemann pronounces to be homoiopathic, or capable of producing in the body of a person in health, the very diseases, which they are so successful in counteracting. If proofs are wanted by any of this position, we may tell them that they must remain in ignorance, for presuming to demand such from our infallible author: or if he deigns to listen to them, he will point to the good effects of applying snow to frost-bitten limbs; and of holding a burned finger to the fire.

We do not propose to enlarge upon the truth of this doctrine; for independently of all sound logical reasoning, we are told that experience, that infallible oracle, stamps it with the seal of her authority. We do not talk of the experience of those who have practised medicine according to the vulgar and erroneous notions which have hitherto prevailed, by prescribing for diseases a farrago of medicines, which would puzzle Æsculapius himself to explain. Fifty years of such experience, are so many spent in looking through a kaleidoscope, which has been constantly revolving; we see an infinity of shapes and of colours; but they pass away and we can give no account of them. A few established facts will demonstrate that it is not in the power of Nature herself to cure an already existing disease, by opposing to it another which is dissimilar in its nature, however intense and severe it may be; and that no remedies, however energetic are of any avail, if they are not homoiopathic. For example, epilepsy has yielded to an attack of tinea of the scalp; but the tinea having been cured, the epilepsy returned; the itch has been driven from the field by scurvy, but speedily re-asserted its claims when the foe retired: typhus fever has suspended phthisis, as long as the symptoms of fever lasted; mania has done the same; when small-pox attacks a patient labouring under measles, the symptoms of the latter are usually arrested, till the former has run its course; scarlatina has ceased on the appearance of the vaccine vesicle. It sometimes indeed happens that the new, or second disease, after having for a long time acted upon and infected the system, forms an alliance with the original one, in spite of their dissimilitude; but in such cases each invader has separate head-quarters and distinct encampments, and his sway is limited to particular districts; thus the pox may become of an itchy nature; and the itch on the other hand may be of a poxy nature; the two diseases being dissimilar, the one cannot in the nature of things annihilate, or cure the other, although the symptoms of syphilis are suspended when an eruption of psora makes its appearance on the skin; but in course of time the two diseases being equally strong and powerful, instead of continuing a useless warfare, establish a reciprocal union, by which they may live in the same house together, each occupying those apartments which please and suit it best. If therefore Nature fails to cure any disease by the addition of another which is of a different nature, how should men be so weak as to expect better success? Yet on such a sandy basis, has the structure of medicine been hitherto attempted to be reared; for what are allopathic medicines, but substances which have the power of exciting a morbid state in the system, in no respect like unto the disease against which they were administered. Purgatives have been prescribed for the cure of the itch; and issues have been ordered in cases of epilepsy; and we will not deny that some benefit has been thus occasionally obtained; but it is only temporary; the germ remains behind, and the baneful weed will soon re-appear.

But the result is very different, when two diseases which are similar, meet together in the system; in other words, when to a disease already existing, there is added a second of the same nature, but of greater energy; the cure is at once speedy and agreeable. Such is the homoiopathic doctrine, in the support and confirmation of which a very few examples will suffice. Variola, one effect of which is often to destroy the vision, has in numerous cases cured obstinate ophthalmiæ; asthma and deafness have yielded to the same; agues have been eradicated by vaccination ; hooping-cough by measles ; obstinate cutaneous eruptions by almost all the exanthemata ; and long and severe asthmas and consumptions, which had owed their origin to a *psoric* taint in the constitution, have vanished as it were by a charm, when a fine crop of itchy papulæ and vesicles appeared on the surface.

It is surprising that these facts had not hitherto opened the eyes of men to the true principles of treating diseases ; and that it has been reserved for a physician of the 19th century to be the interpreter of such simple and sublime laws ; after such convincing evidence it is impossible for any rational medical man to persist in the absurdities of allopathia, and of enantiopathia ; at least so says Dr. Hahnemann :—it will be necessary to wait a little, before all be so thoroughly convinced of their former errors as the Doctor.

Medical men have been misled, he thinks, by considering the human body as a laboratory, or place for chemical experiments, vainly and foolishly supposing that they could neutralise diseases, as they do acids by adding alkalis ; or alkalis by adding acids ; but we need not detain our readers any longer on this section of the book ; and we shall therefore proceed to develop a few of the very original ideas of our author on various diseases. These he divides into acute and chronic ; and of the former class he enumerates two orders, distinguished from each other by the difference of their exciting causes, and by their occurring occasionally and sporadically ; or as epidemics and pestilences. The former are excited sometimes by improper diet, by passions of the mind, by the vicissitudes of the weather, by fatigue, &c. &c. but far more frequently by "*recrudescenses passagères*" of a latent psoric affection ! ! whereas the latter are dependent either on atmospheric and terrestrial influences, or on the miasmata of contagion.

The second or chronic class of diseases is thus defined.

"They are all the morbid states which give rise to a chronic miasm ; their nature is such, that if not opposed by homoiopathic remedies, they inevitably become worse and worse, till they induce the death of the patient." There are only three essential and original chronic diseases, syphilis, sycosis, and psora, of which the last is by far the most important, and most prolific of distress to mankind ; it is the true exciting cause of hysteria, hypochondriasis, mania, epilepsy, convulsions, and spasms of all sorts, rachitis, cancer, gout, dropsies, hæmorrhages, asthma, diseases of the eye, palsy, gravel, impotency, sterility, and a thousand other ills, to which poor man is subjected in this world below. Now as all these maladies are the effects of one poison, the treatment becomes easy, efficacious and rational. We need not therefore say much on this head ; but refer the reader, who no doubt will be curious to know more about this " grande verité " to another and a still more voluminous work by the same author, " Traité des Maladies Chroniques."

After this, let us hear no more of the pompous nosologies of Sauvages, Cullen, and Mason Good ! they have imposed their systems on the world too long : the classification of all diseases is now as simple as their cure. More cannot be said of the infinite superiority of the homoiopathic doctrine ; it is only requisite to understand how to put its principles and precepts in practice. A few words will suffice to illustrate this part of our subject. Let us take the case of insanity ; this is one of the offspring of the psoric pollution ; and the ratio medendi is, to exhibit antipsoric medicines, which are enumerated and explained at the end of the book. Intermittent fevers are to be treated in the same manner. Fortunately the Dr. has published the details of two cases, which we gladly present to the readers, as we must frankly confess that we should have been somewhat puzzled to have given an adequate description of the new system of therapeutics.

*Case* 1. S. A. aged 40, had the following symptoms :—

1. Pungent pains at the scrobiculus cordis, on any motion of the body ; felt especially on rising up, and on making any "*faux-pas.*"

2. No pain, nor uneasiness when she was in the horizontal position.
3. Could not sleep at any time, till it was eight o'clock in the morning.
4. Had a good appetite; but as soon as she eat food, she experienced uneasiness at the heart.
5. Another effect of eating was to cause the flow of a great quantity of saliva, which trickled from her lips.
6. After every time that she eat, she experienced at intervals ineffectual retchings.
7. Her character was violent and passionate—she sweated copiously on suffering any pain!!
8. In all other respects, her health was normal.

We have detailed the case as it is reported, to exhibit the proper method of describing diseases, and also with the view of shewing how beautifully and aptly the principles of homoiopathia will apply. Now, a variety of different medicines will produce the individual symptoms of the above case, but only one has the property of giving rise to every one of them; we are told, for example, that belladonna, cinchona, rhus toxicodendron, occasion prickings at the pit of the stomach; but not as with our patient *only* at those times when she moved about. The anemone pulsatilla, however, has the power of causing this symptom, when a person stumbles or makes a false step; but, unfortunately, it cannot disorder the digestion, nor make people bad-tempered, as is mentioned to have been the case, vide No. 4, 5, 6, and 7 of the catalogue of symptoms. In short, the "bryonia" is the only herb known which will answer our purpose.

In the same manner, we must carefully consider and investigate each symptom separately, and try to find out the homoiopathic remedy. The briony is such, for when given to a person in health, it will occasion every bodily and moral inconvenience enumerated above; and, therefore, it must necessarily and inevitably cure these self-same inconveniences when they are the result of disease. Having thus discovered the proper remedy, the next thing to be attended to is the dose; and as the patient was robust and vigorous, a most powerful homoiopathic dose was exhibited! Reader, do not be astonished when we tell you, that no less than one undiluted drop of the juice of the herb was given; and so rapid were its effects, that in 24 hours she had quite recovered.

*Case* 2.—A man, aged 42, pale and feeble, had been ill for five days. The symptoms were:—
1. At first vertigo and sickness, with frequent retching.
2. On the second night, at 2 o'clock, vomitings of a sharp acid matter.
3. On the succeeding nights, violent and distressing retching.
4. On the fifth day, he was much annoyed with a disagreeable taste in the mouth, and with offensive smells.
5. He felt as if the food lay undigested in his stomach.
6. His head was confused, and his mind irritable.
7. The slightest noise vexed him.
8. Temper mild, patient, and not easily ruffled.

Different medicines have the power of exciting one or more of the above symptoms; but the anemone pulsatilla alone can excite them all at the same time, and must, therefore, be considered as the correct homoiopathic remedy for the case. The dose ordered was half a drop of the four-millionth part of a strong drop of the expressed juice. The patient was completely cured in less than a day.

After perusing these two reports, the reader will not be surprised to hear the following announcement.

"Whenever the exhibition of a homoiopathic medicine has been judiciously made, the disease for which it is prescribed, if it be of recent origin, vanishes in a few hours—if it has existed longer in the system, the time required for a cure will not exceed a very few days, and health is restored by a transition at once rapid and insensible ;" thus "belladonna" is a specific against scarlatina, both as a preventive and as a curative; "aconitum napellas" against purpura; "burnt sponge" and "sulphuret of lime" against croup ; "drosera rotundifolia" against hooping-cough ; "corrosive sublimate," in doses of about a twenty-millionth part of a grain, against dysentery, which may be thus cured instanter; and the "thuya occidentalis" against a disease which hitherto never has been cured, we mean sycosis ! ! ! "These incontestible proofs of the truth of my doctrine," says Hahnemann, "cannot be more shaken by the malicious attacks, however virulent, of physicians educated in prejudices, and taught to follow the routine of the schools, than the immortal discovery of Harvey was by the base calumnies of Riolan."

The last section of the present work is occupied with a detail of the homoiopathic pharmacopœia, enumerating the drugs, the mode of preparing them, their doses, antidotes, and other particulars. We select cinchona, to furnish our readers with some knowledge of this part of the subject. The author confirms the vulgar opinion, that it may be considered almost as a specific in ague ; for he has discovered that it is capable of inducing all the symptoms of this disease, if it be given to a person in health. The large doses usually administered are severely reprobated ; he finds that one drop of a tincture so prepared, as to contain only the $\frac{1}{1000000000000000000000000}$th (we are not skilful enough in arithmetic to be able to designate the fraction) of a grain is a dose; which is often too powerful, and always sufficient to produce the special effects of the drug, and that it is very rarely necessary to repeat the dose ; but it may very naturally be enquired, how are such infinitesimal divisions to be obtained ; the method is simple and easy—take one grain of the medicine, and rub it very carefully together with 100 grains of sugar; then take a grain of this mixed powder, and rub it with another 100 grains of sugar, and so on, till you bring it to what our author terms the millionth power, that is, till the prepared powder contains a millionth part of a grain. On one grain of this pour a hundred drops of proof spirit, and dissolve it by shaking the bottle with two scientific turns of the fore-arm. A drop of this tincture is then to be added to 99 drops of alcohol, and shaken twice as before ; and the dilution may be thus carried to any extent ; but so active are some medicines, that even after such a division as defies mere words to express by language, it is found necessary to prescribe only the 100th part of a drop ! ! The only specimen of a formula in the whole work is very imperfect: but we shall transcribe it literally.

℞. Belladonnæ . . . . . . . x. (un décillionième) 2-8th (un
dix milleoctillionième)
Globuli saccharini . . No. ij. iij. iv. vj. x. &c. &c.
Sacchari lactei . . . . gr. iij. Misce exacte.

With a single reflexion we shall now close this notice of the work before us ; and we enquire when will vain, conceited man be satisfied with observing facts as they are presented to his eyes, and employing his memory to treasure and his judgment to arrange them ! Is not this a sufficiently ample task, and one which is sufficiently noble for him ? Why does he go on to turn and twist the simplest truths to suit some idle fancy of his brain ? why does he bend what is straight ? why does he darken what is clear ? The humblest and homeliest mind, unfettered by prejudice, will often make greater progress in the search after truth than he who has been educated in all the mysteries of scholastic wisdom ; for the one is content to plod his way on the great high road, while the other, seduced by a flower or a butterfly, loses himself in the entanglements of some byepath. We cannot too urgently impress upon all medical men the importance of collecting much and building little ;—at best ours is an uncertain science ; and its uncertainty must be a thousandfold increased by the spirit, now so much abroad, of its votaries, struggling to frame some cobweb hypothesis from partial and insulated facts. Is it not far more worthy of a philosophic mind diligently and studiously to examine Nature's ways; to note down facts as they occur; to mark how they stand to each other, whether they are uniform in their precedence and sequence, or whether they appear to follow each other accidentally; to assemble all and exclude none; and when we attempt, which ought, however, to be seldom, to arrive at the discovery of a general truth, or, what is foolishly termed in medicine, a proximate cause, to deduce it from a host of phenomena observed at different times, in different places, and by different men. It is from want of attention to this maxim of true wisdom, that every petty mason of our craft aspires to be a master architect, and thus, instead of one great, and solid, and solemn edifice for the enshrinement of our goddess, there are numberless puny hovels, confusedly scattered—reared one day to be blown down the next. "The object of medicine," we use the words of our author, who, while he himself indulged in such fantastical and whimsical reveries, nevertheless could see the absurdity and error of others, "does not consist in framing systems out of empty ideas and hollow hypotheses on the essence of the vital principles, and on the production of diseases, or in vainly seeking to explain morbid phenomena, by drowning all common sense in an eddy of unintelligible words and by a parade of useless observations and experiments, which may impose on the ignorant, but will not relieve the sufferings of the distressed. We have enough of these scholastic follies, dignified by the name of theory of medicine, and enshrined in the chairs of universities and colleges. It is now high time, that whoever calls himself a physician should cease to deceive mankind by unmeaning jargon, and begin to act—to mitigate woe, and to effectually cure disease."

